# Supplementary material for: Transcriptional Differences for COVID-19 Disease Map Genes between Males and Females Indicate a Different Basal Immunophenotype Relevant to the Disease
Source: Genes (Basel). 2020 Dec 1;11(12):1447. doi: 10.3390/genes11121447 (PMC7761414; doi:10.3390/genes11121447)

Supplementary Figure 1: The Principal Component Analysis plot for GTEX RNA-seq data of immune tissues/cells, including whole blood, spleen, and lymphocytes. Each dot corresponds to an individual. The cause of death is indicated in the label. **a** Whole blood. PCA shows that the samples labeled as "ventilator case" strongly clustered apart from the rest of the first PC samples. These samples were, therefore, excluded from the DeCovid app**. b** Spleen. No bias is observed and all samples were included in the DeCovid app. **c** Lymphocytes. No bias is observed and all samples were included in the DeCovid app.

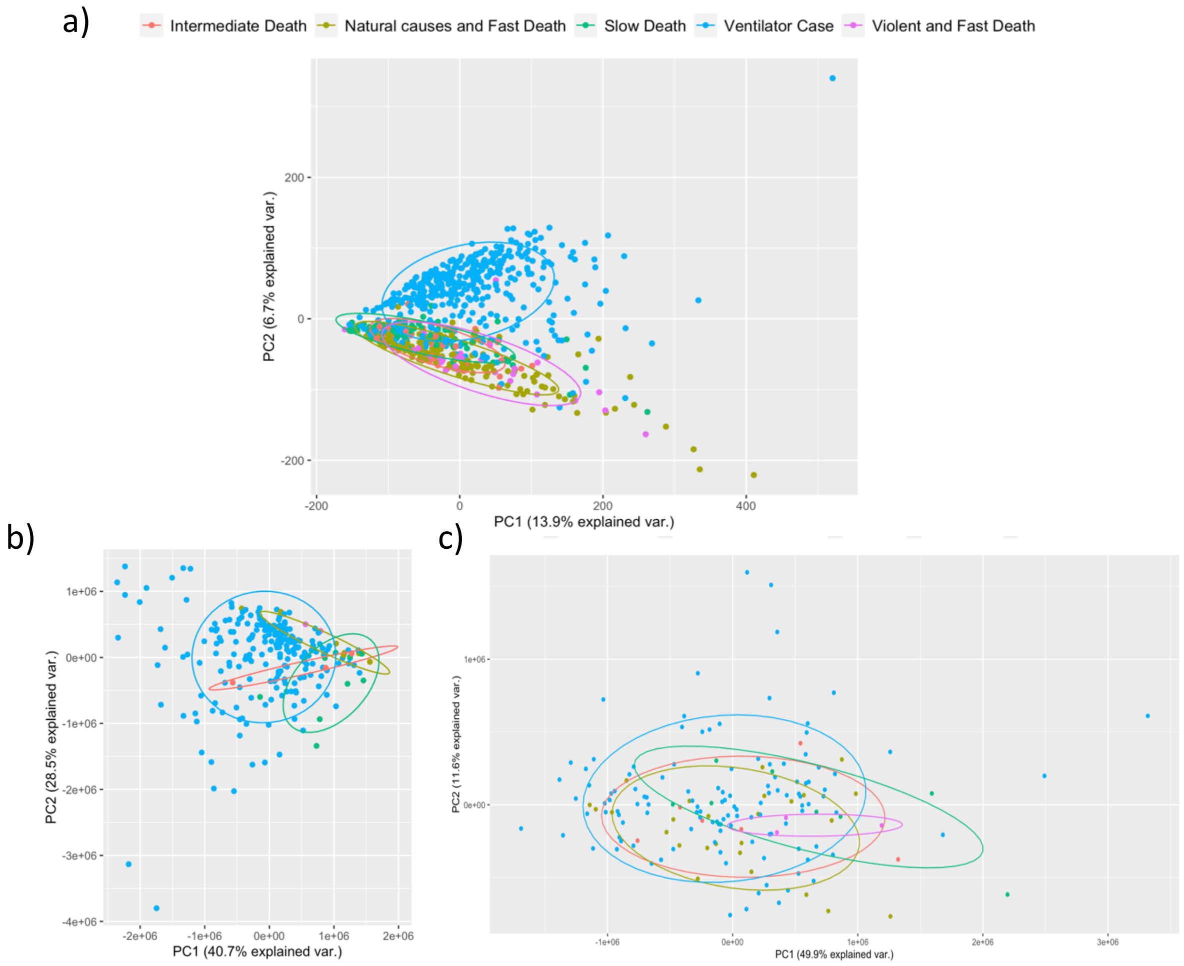

Supplementary Figure 2: Heatmap with average sex and age significant expression differences for selected COVID-19 genes. Results for different immune system tissues and cell types and shown.

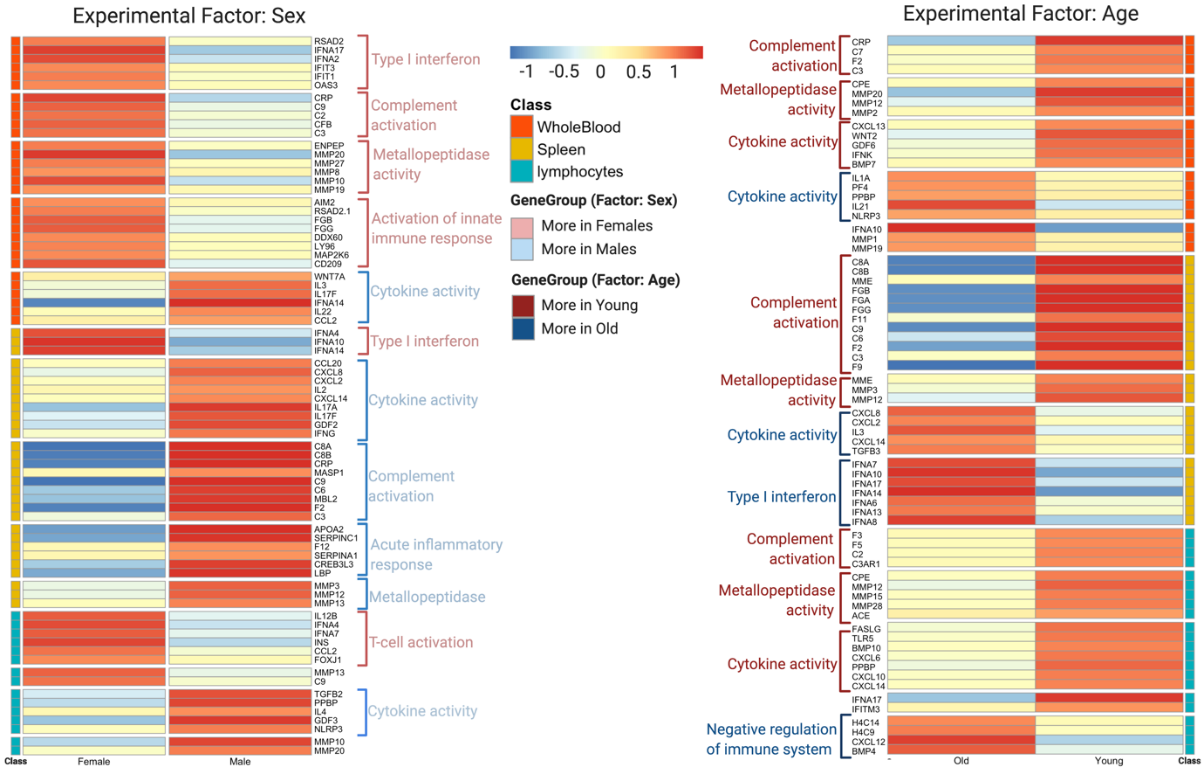

Supplement: Supplementary file 1 [file genes-11-01447-s001.zip › supplementary materials/Supplementary_Figures2.pdf]
