# Supplementary material for: Transcriptional Differences for COVID-19 Disease Map Genes between Males and Females Indicate a Different Basal Immunophenotype Relevant to the Disease
Source: Genes (Basel). 2020 Dec 1;11(12):1447. doi: 10.3390/genes11121447 (PMC7761414; doi:10.3390/genes11121447)

Supplementary Material: Instructions for running DeCovid.

DeCovid: A tool for conducting a Wide-spread transcriptional analysis for COVID-19 Disease Map genes

How to Run DeCovid

There are two main options to run DeCovid:

1. Run DeCovid Docker image
2. Install and run as *R package*.

Run DeCovid Docker Image

1. Requirements:
   - Docker engine.

2. Run Docker image in **terminal** (Mac/Linux) **PowerShell** (Win):

```
docker run --rm -p 3838:3838 conesalab/decovid:decovid
```

3. Open the URL that the **terminal** (**PowerShell**) will return (typically http://[::]:3838) in a **web browser**.

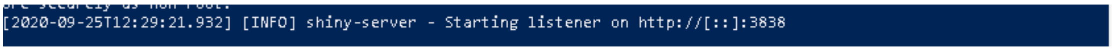

Run DeCovid Shiny App

1. Requirements:
   - RStudio
   - Install following R packages:

```
install.packages("shinydashboard")
install.packages("shiny")
install.packages("pheatmap")
install.packages("ggplot2")
install.packages("png")
install.packages("grid")
install.packages("DT")
install.packages("scales")
BiocManager::install("edgeR")
BiocManager::install("clusterProfiler")
BiocManager::install("org.Hs.eg.db")
```

2. Download Code and files in GitHub:
   - https://github.com/ConesaLab/DeCovid
3. Open **app.R** file and click Run App

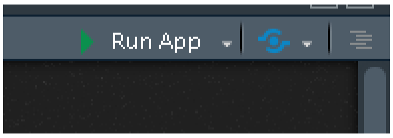

Supplement: Supplementary file 1 [file genes-11-01447-s001.zip › supplementary materials/Supplementary_Materials_Installation.pdf]
